# Editorial: Cell Signaling in Host–Pathogen Interactions: The Host Point of View

**DOI:** 10.3389/fimmu.2018.00221

**Published:** 2018-02-19

**Authors:** Diana Bahia, Abbhay R. Satoskar, Olivier Dussurget

**Affiliations:** ^1^Departamento de Biologia Geral, Instituto de Ciências Biológicas, Universidade Federal de Minas Gerais, Belo Horizonte, Brazil; ^2^Department of Pathology, Wexner Medical Center, The Ohio State University, Columbus, OH, United States; ^3^Department of Microbiology, The Ohio State University, Columbus, OH, United States; ^4^Unité des Interactions Bactéries-Cellules, Institut Pasteur, Paris, France; ^5^Institut National de la Santé et de la Recherche Médicale, U604, Paris, France; ^6^Institut National de la Recherche Agronomique, USC2020, Paris, France; ^7^Université Paris Diderot, Sorbonne Paris Cité, Paris, France

**Keywords:** host–pathogen interactions, signaling, innate immunity, kinase, evasion mechanism

**Editorial on the Research Topic**

**Cell Signaling in Host–Pathogen Interactions: The Host Point of View**

What is a host? Answering this simple question is a complex task according to Casadevall and Pirofski (1), who stated that “a host is an entity that houses an associated microbiome/microbiota and interacts with microbes, such that the outcome results in damage, benefit, or indifference, thus resulting in the states of symbiosis, colonization, commensalism, latency, and disease.” What is a host in the context of a host–pathogen relationship? Considering that pathogenicity is a microbial property that occurs in a susceptible host, the host is an entity that houses its own microbiota and interacts with pathogenic microorganisms. The outcome of this interaction is a trade-off between host, microbiota, and pathogen (2).

The initial pathogen–host interaction involves the recognition of conserved microbial components known as the pathogen-associated molecular patterns (PAMPs) by the host pattern recognition receptors (PRRs). As the first line of host defense, this PAMP-PRR interaction is a critical determinant of the success or failure of an immune response (3). The PRRs are present at the cell surface or intracellularly in many cells of the innate immune system, including epithelial cells, macrophage-monocytes, granulocytes, mast cells, and dendritic cells (DCs) (4).

Following PAMP-PRR binding, signal transduction initiates a complex cascade of cellular reactions, leading to an early host response that not only contributes to pathogen elimination, but also represents an important link to the adaptive immune response (5). Cellular reactions include the activation of kinase pathways and production of effector molecules, which in turn activate transcription factors and induce cytokine gene expression that ultimately modulates the innate immune system to a proinflammatory or anti-inflammatory response (6). Major signal transduction cascades of the host immune response, such as the Janus kinase/signal transducer and activator of transcription, the nuclear factor kappa-light-chain-enhancer of activated B cells (NF-kB), the mitogen-activated protein kinase (MAPK), and the phosphatidylinositol-3-kinase pathways, have been unraveled over time (7).

This research topic provides recent findings on how the cell signaling machinery is activated in its tight communication with pathogens and focuses on the cellular signaling mechanisms that are essential for host immunity and their subversion by pathogens.

This special issue is a collection of timely, high-quality invited articles. It includes reviews and opinion articles, which discuss the recent progress in our understanding of host defense components, in particular, the innate immune receptors, autophagy and organelles, signaling cascades, and immune signaling. This issue also includes original articles that discuss new aspects of the host immune response using various *in vivo* and *in vitro* models of infection. These findings pave the way for the discovery of novel host and/or pathogen targets, which could lead to the development of innovative anti-infective therapies.

## Review and Opinion Articles

### Receptors

Purinergic receptors detect extracellular adenosine and adenosine triphosphate that activate intracellular signaling events and are ubiquitously expressed in mammalian cells. Swartz et al. reviewed the role of purinergic receptors as an important link between HIV infection and chronic inflammatory responses. The SLAM family (SLAMF) of receptors are cell surface glycoproteins, which represent another type of receptor that acts as a microbial sensor. SLAMF receptors play critical roles in regulating innate and acquired immune responses. van Driel et al. discussed how SLAMF receptors and their specific adaptors regulate immunity against pathogens.

Microbial pathogens have evolved multiple mechanisms to evade immune recognition by PRRs and interfere with receptor signaling to promote infection. McGuire and Arthur reviewed the effectors produced by pathogenic bacteria to subvert toll-like receptor signaling, with a focus on proteins that target the MAPK and NF-kB pathways. Sensing of microbe-associated molecular patterns and danger-associated molecular patterns by cell surface PRRs play a key role in the activation of the immune response and inflammation. As important is the sensing of pathogens and endogenous molecules by a growing number of cytosolic sensors. Radoshevich and Dussurget highlighted the most recent discoveries in the field of nucleic acid cytosolic sensing and review the mechanisms evolved by pathogens to subvert host cytosolic surveillance.

### Autophagy and Organelles

Autophagy is a self-catabolic process that plays a critical role in the clearance of bacterial, viral, and parasitic pathogens. Conversely, intracellular pathogenic microbes, such as mycobacteria, inhibit autophagy for their survival. Escoll et al. discussed how autophagy modulators could be used as novel host-directed drugs for the treatment of infectious diseases. Kanayama and Shinohara reviewed recent discoveries on the role of host autophagy in antifungal immunity.

The integrated endoplasmic reticulum stress response (IERSR) is critical for cell survival in response to stress. It is becoming increasingly evident that IERSR contributes to the pathogenesis of infections caused by bacterial pathogens. Dias-Teixeira et al. discussed the role of IERSR in the intracellular survival of the pathogenic protist *Leishmania*. Understanding how the IERSR promotes the survival of *Leishmania* could lead to the development of novel host-directed therapies against leishmaniasis.

### Signaling Cascades

Protein kinases regulate critical cell functions. Soares-Silva et al. reviewed the MAPK activation of cells upon *Leishmania* sp. infection. Zhang and Kima reviewed other host cell protein kinases that are activated during *Leishmania* infection of mammalian cells, such as the non-receptor protein kinase Abl family and the protein kinase regulated by RNA (PKR). MAPK host cell modulation and immune evasion by another intracellular parasite, *Trypanosoma cruzi*, is reviewed by Soares-Silva et al. Watanabe Costa et al. described how molecules secreted by *T. cruzi* modulated different signaling pathways in mammalian cells. Keating and McGargill focused on the emerging and sometimes contradictory roles of mTOR in orchestrating lymphoid cell-mediated host immune responses to pathogens. Mukherjee et al. reviewed how the reciprocal action of protein kinase C (PKC) isoforms regulated the immune response to cellular pathogen infection by the regulation of protein kinases.

### Immune Signaling

The cells of the innate immune system, such as the phagocytes, play a critical role in mediating the early resistance against microbial pathogens. However, it is becoming increasingly evident that lymphoid cells, including CD4+ T cells, also display an innate-immune cell-like phenotype. A review by Cruz-Adalia and Veiga summarized the current understanding of the innate-like behavior of lymphocytes. LaRock and Nizet highlighted the role of IL-1β/inflammasome signaling during infections caused by streptococcal pathogens. Guignot and Van Nhieu reviewed host cell responses to transient pore formation induced by the type III secretion system of Gram-negative pathogenic bacteria. Correia et al. highlighted the importance of post-transcriptional regulation of gene expression mediated by intracellular miRNAs in mammalian infection and immunity processes.

Leprosy remains a significant global health problem. Erythema nodosum leprosum (ENL) is a complication of leprosy, but the mechanism of its pathogenesis is not clear. A review by Polycarpou et al. summarized the immunological studies on ENL, which may enhance our understanding of the pathogenesis of this disease. Infections caused by *Trypanosoma brucei* are responsible for significant morbidity and mortality globally. These parasites survive within the host by evading host immunity through the manipulation of host immune functions. Kuriakose et al. summarized the effect of *Trypanosoma* on signaling pathways and cytokine production by macrophages. Gannavaram et al. focused on the polarization of antigen-presenting cells and the subsequent role of costimulatory and coinhibitory molecules in mediating vaccine-induced immunity using live-attenuated *Leishmania* parasites as a specific example. Di Genova and Tonelli reviewed host-protozoan interactions during infection by the intestinal protozoa *Giardia lamblia, Cryptosporidium parvum*, and *Entameba histolytica*, with a focus on the modulation of tight junctions and the cytoskeleton, host gene expression, and apoptosis.

## Original Research Articles

### *In Vivo* Immune Responses

#### Mouse Model of Infection

Most bacterial infection studies are classically performed using planktonic pathogens. Although biofilms are recognized as a major cause of recurrent and relapsing infections, our knowledge of the specific characteristics of bacteria released during biofilm disassembly is still limited. Using a mouse model of *Staphylococcus epidermidis* hematogenous infection, França et al. compared host gene expression, cytokine production, and organ colonization of planktonic, biofilm, and biofilm-released bacteria. The authors showed that the bacteria released from *S. epidermidis* biofilms are characterized by their capacity to trigger a strong inflammatory response.

The facultative intracellular bacterial pathogen *Listeria monocytogenes* has long been used as a vector to develop vaccines. More recently, *L. ivanovii*, a pathogenic species for animals, but generally not for humans, has been used as a live vaccine vector. To better characterize the immune response to an *L. ivanovii* infection, Zhou et al. compared the cytokine production and organ colonization of mice inoculated intravenously or intranasally with *L. ivanovii* or *L. monocytogenes*. The authors showed that *L. ivanovii* is capable of inducing a cytokine response similar to that of *L. monocytogenes* in the liver after intravenous inoculation and in lungs after intranasal inoculation, but caused milder organ damage.

#### Other Hosts

Duck Tembusu virus is a newly emerging virus that can cause an acute contagious infection in ducks that is characterized by reduced egg production. Li et al. systematically investigated the expression of immune-related genes in the duck spleen and brain and saw the expression of proinflammatory cytokines early in the infection.

The importance of gut microbial community (microbiota) in immune system homeostasis and health has become increasingly evident (8). In addition to regulating the local intestinal immune system, microbiota can have a profound influence on systemic immune responses. By feeding goats with a high concentrate diet (i.e., excessive amounts of non-structural carbohydrates and highly fermentable forages) for short- and long-term, Hua et al. observed alterations in ruminal microbiota and their metabolites, and significant differences in pro- and anti-inflammatory cytokine expression.

A *Streptococcus suis* infection can increase vascular permeability and lead to the potentially lethal streptococcal toxic shock syndrome in humans. Chen et al. investigated the heparin-binding protein (HBP)-dependent vascular leakage triggered by an *S. suis* infection. The authors showed that suilysin (SLY), an exotoxin secreted by *S. suis*, is responsible for the HBP release by neutrophils, and vascular leakage. HBP induced by SLY was related to toll-like receptor 4, p38 MAPK, and the phosphatidylinositol 3-kinase pathway, and dependent on a G protein-coupled seven-membrane spanning receptor.

### *In Vitro* Immune Responses and Cellular Signaling

#### Epithelial Cells

Cytokines of the IL-36 family are known for their role in mediating inflammation and host defense. Among them, IL-36 gamma has the highest expression levels in damaged epithelium, suggesting that it plays a role in the epithelial immune response. The IL36 receptor is a crucial mediator molecule in the inflammatory response. By exposing vaginal and endocervical epithelial cells to microbial products to measure innate inflammatory responses, Winkle et al. demonstrated for the first time the induction and regulation of IL-36 gamma and its receptor in the human female reproductive tract and showed that they are differentially induced by microbial products at this site.

Probiotics are beneficial bacteria that when administered in adequate amounts caused a health benefit for the host. It is believed that probiotics are effective in cancer therapy, mainly because of their positive effects on the immune system. Nami et al. tested the effectiveness of the probiotic *Enterococcus lactis* IW5 obtained from the human gut against various cancer cells. In addition to strongly inhibiting the growth of several pathogenic bacteria, *E. lactis* IW5 secreted products that decreased the proliferation and viability of all cancer cell lines and triggered apoptosis of cancer cells possibly *via* the extrinsic IL-3 receptor pathway.

The pathogenic fungus *Histoplasma capsulatum* is the etiological agent of histoplasmosis, which primarily affects the lungs or other organs in a disseminated form. Maza and Suzuki investigated cytokine secretion by human lung epithelial cells upon infection with *H. capsulatum*. The authors showed that *H. capsulatum* induced IL-6 and IL-8 production after recruitment of α3 and α5 integrins to the membrane rafts and activation of the Src-family kinase. *Candida albicans* is a commensal fungus that can eventually lead to a hematogenic infection, once they invade cells and reach deep tissues. Maza and Bonfim-Melo et al. showed that the ability of *C. albicans* to invade HeLa cells was dependent on Src-family kinases, and *C. albicans* resistance to oxidative stress was not linked to hyphal length.

#### Phagocytic Cells

Mast cells express vascular endothelial growth factor (VEGF), which implicates them in pro-angiogenic processes. Johnzon et al. demonstrated that *S. aureus* could induce VEGF in mast cells, which was partly dependent on the NF-κB pathway.

Song et al. demonstrated that *Vibrio fluvialis*, which causes human diarrhea, secretes hemolysin. Hemolysin induced cytotoxicity and the secretion of IL-1β through the activation of the NLRP3 inflammasome in macrophages and contributed to the inflammatory pathology in the colon of mice.

The receptor-interacting serine/threonine kinase 2 (RIPK2) is an important activator of NF-κB and plays a role in the immune response and apoptosis. Wex et al. identified the enzyme CYLD as an inhibitor of RIPK2. The authors showed that CYLD deubiquitinated RIPK2 in macrophages infected with *L. monocytogenes*, leading to impaired activation of NF-κB, reduced production of proinflammatory cytokines and reactive oxygen and nitrogen species, which ultimately resulted in impaired infection control.

Macrophages play a key role in the immune response to pathogen invasion. They can be polarized into two distinct phenotypes: M1 macrophages induced by T helper 1 (Th1) cytokines and M2 macrophages induced by Th2 cytokines. Helminths have coevolved with their hosts and they survive in them as long as possible, reaching a state of tolerance. Helminths have developed mechanisms to subvert the immune system, such as secretion and/or excretion of protein products that will impact the macrophage polarization type. Zawistowska-Deniziak et al. used THP-1 macrophages stimulated with a *Hymenolepis diminuta* (HD) tapeworm and its excretory/secretory products (ESP). They identified the anti-inflammatory properties of HD (i.e., inhibition of TNF-α, IL-6, and IL-1β after ESP stimulation), while the whole living parasite induced proinflammatory cytokines. Additionally, ESP and HD led to different phosphorylation profiles in macrophages. Therefore, HD and ESP induced a mixed polarization of macrophages.

MicroRNA have emerged as key regulators of innate immunity and modulate the ability of DCs to present antigens and secrete cytokines (9). Lin et al. showed that the influenza virus could secrete PB1, a protein that downregulates the expression of miR375, which enhanced the function of DCs. This effect was accomplished by the inhibition of the JNK signaling pathway and the activation of the ERK signaling pathway.

Osteoclast differentiation is driven by two cytokines, cytokine receptor activator of NF-κB ligand (RANKL) and macrophage colony-stimulating factor. The toxin produced by *Pasteurella multocida*, PMT, mediates RANKL-independent osteoclastogenesis (Chakraborty et al.). PMT led to the differentiation of bone marrow-derived macrophages into functional osteoclasts. PMT-mediated induction of IL-6 and TNF-α, but not IL-1, supported the differentiation process.

We believe that the excellent reviews and the cutting-edge contributions of this research topic advances our understanding of host signaling in response to infection and provided new insights into the booming field of host–pathogen interactions.

## Author Contributions

DB, AS, and OD conceived and wrote the manuscript.

## Conflict of Interest Statement

The authors declare that the research was conducted in the absence of any commercial or financial relationships that could be construed as a potential conflict of interest.
